# Reply to Alberts, P. Comment on “Hietanen et al. Cytolytic Properties and Genome Analysis of Rigvir^®^ Oncolytic Virotherapy Virus and Other Echovirus 7 Isolates. *Viruses* 2022, *14*, 525”

**DOI:** 10.3390/v14092078

**Published:** 2022-09-19

**Authors:** Eero Hietanen, Petri Susi

**Affiliations:** Institute of Biomedicine, University of Turku, Medisiina D, Kiinamyllynkatu 10, 20520 Turku, Finland

Our work focused on comparing the cellular effects of Rigvir to other echovirus 7 isolates, because originally Rigvir is also an echovirus 7 isolate. In addition, we used the genome sequences made by us and others to put Rigvir on a phylogenetic map of echovirus 7 isolates. In his comments, Dr. Alberts aims to clarify the actual state of Rigvir as a potential oncolytic virus in clinical use from company perspective [1]. We admit that his comments correctly declare that our paper does not include any clinical data. Furthermore, he claimed that our data ignore previous cellular results, but in fact, previous cellular studies prompted us to perform the current study. By using other E7 isolates as “controls” for Rigvir, we aimed to demonstrate that the performance of Rigvir is not that different from the other clinical echovirus 7 isolates after all, and therefore, it is duly justified that its performance should be (re)-evaluated against related viruses. Dr. Alberts also questions the origin of our Rigvir batch. We obtained the virus from a colleague who purchased the virus ampules from a company representative for the treatment of a relative suffering from terminal cancer. The ampules are, indeed, as described by Dr. Alberts and were stored at recommended temperature at all times, and therefore, we have no reason to doubt their origin. In fact, we would be rather alarmed if falsified Rigvir ampules were available on the market. If that would be the case, we would not expect to isolate echovirus 7 or its sequences from the ampules. We regret if our descriptions have been inaccurate. We do not dispute the finding that Rigvir may possess cytolytic potential, which is also brought up in the original publication, along with additional citations to studies examining Rigvir’s ability to reduce the viability of various carcinomas. Instead, we call for further studies regarding Rigvir’s efficacy, especially regarding what potentially makes it unique compared to clinical E7 isolates, which possess a seemingly similar infection profile across native and cancer cell lines. It is our understanding that clinical trials performed with Rigvir are small in terms of the number of trials and in the number of test subjects. Dr. Alberts refers to earlier clinical studies performed in 1968–1991. However, as we wrote, these data are either difficult to obtain, they are written in Russian, or simply do not reach the current standards for clinical use within the EU area. In addition, our opinion is that future studies should also address the mode-of-action of Rigvir. Finally, we appreciate the possibility that the marketing information is constantly being updated on the company’s website.
